# Central Pain Mimicking Trigeminal Neuralgia as a Result of Lateral Medullary Ischemic Stroke

**DOI:** 10.1155/2019/4235724

**Published:** 2019-10-27

**Authors:** Abinayaa Ravichandran, Kareem S. Elsayed, Hussam A. Yacoub

**Affiliations:** ^1^Lehigh Valley Physician Group – Neurology, Lehigh Valley Health Network, Allentown, PA, USA; ^2^Morsani College of Medicine, University of South Florida, Tampa, FL, USA

## Abstract

**Background:**

Central pain mimicking trigeminal neuralgia (TN) as a result of lateral medullary infarction or Wallenberg syndrome has been rarely reported.

**Case Report:**

We discuss a patient who presented with a lateral medullary infarct and shortly after developed facial pain mimicking TN. We also elaborate on the anatomical pathway of the trigeminal nerve explaining facial pain as a result of a lateral medullary lesion.

**Discussion:**

Clinicians should be aware of this typical complication of lateral medullary infarct in order to attain proper management and work-up.

## 1. Introduction

Trigeminal neuralgia is a common form of facial pain that is characterized by paroxysmal and excruciating pain along the distribution of the trigeminal nerve. Although this condition encompasses diverse etiologies, the International headache society has separated classical (or idiopathic) TN from the symptomatic form depending upon the presence of a structural lesion [1]. Increased utilization of magnetic resonance imaging (MRI) offers extensive evidence that most classical cases of TN are found to be associated with compression of the dorsal nerve root by tortuous or aberrant vessels (IHS) [1]. Space-occupying lesions including tumor, multiple sclerosis, and stroke account for 15% of all cases of TN [2]. Facial pain as a result of lateral medullary infarct can mimic TN with a wide reported incidence of 9% (Sacco et al.) [3] to 83% (Merritt and Finland) [4].

In this manuscript, we discuss a case of a patient who developed classic symptoms of central pain mimicking TN shortly after a lateral medullary infarct was diagnosed.

## 2. Case Report

A right-handed 81-year-old man with a past medical history of ischemic stroke, hypertension, hyperlipidemia, type-II diabetes mellitus, and paroxysmal atrial fibrillation presented to the hospital with symptoms of unsteadiness, mild dysphagia, and sharp stabbing right-sided facial pain. Neurological examination revealed decreased pin-prick sensation on the right side of the face. The patient had normal strength bilaterally. Coordination examination showed finger-to-nose dysmetria bilaterally, but more pronounced on the right. Ataxia with swaying to the right was observed on gait examination.

**Figure 1:**
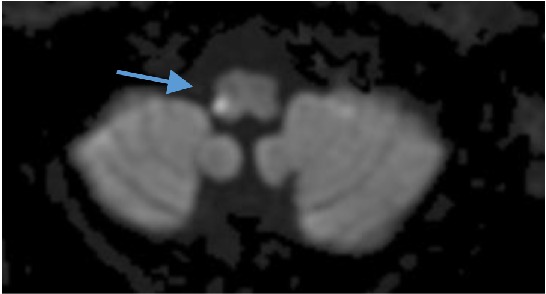
Diffusion weighted MRI demonstrating an area of restricted diffusion in the right posterior lateral medulla (arrow), consistent with an acute infarct.

MRI of the brain revealed an area of restricted diffusion within the right posterior lateral medulla consistent with an acute or subacute infarction (Figure 1). Coagulation studies revealed an international normalized ratio of 2 while on warfarin; the patient endorsed some noncompliance with the recommended regimen.

The patient noted the concomitant onset of right “sharp-stabbing” intermittent pain merely hours after the onset of unsteadiness. The pain occurred in the ophthalmic and maxillary distribution of the trigeminal nerve and was acutely worsened by cold temperature. He was diagnosed with central facial pain secondary to lateral medullary infarct. The pain initially improved with the administration of carbamazepine, which was discontinued after hospital discharge due to adverse reactions. He was subsequently started on gabapentin 100 mg by mouth three times a day with some relief, but stopped the medication secondary to an adverse reaction of dizziness. The patient still experienced recurrent episodes of right-sided facial pain. Several months after the initial event, he was evaluated in the office and was found to have a nonfocal examination. He continues to complain of brief episodes of right-sided lancinating pain in the forehead, cheek, nose, and periorbital region occurring on average three times a day. Symptoms were aggravated by cold temperature. The patient denied any associated symptoms of lacrimation or sweating.

## 3. Discussion

Central pain, by definition, is caused by lesions of the brain and spinal cord. Central pain caused by a brain stem infarct is well known and has been described since 1997, but published cases in literature are sparse [5].

**Figure 2:**
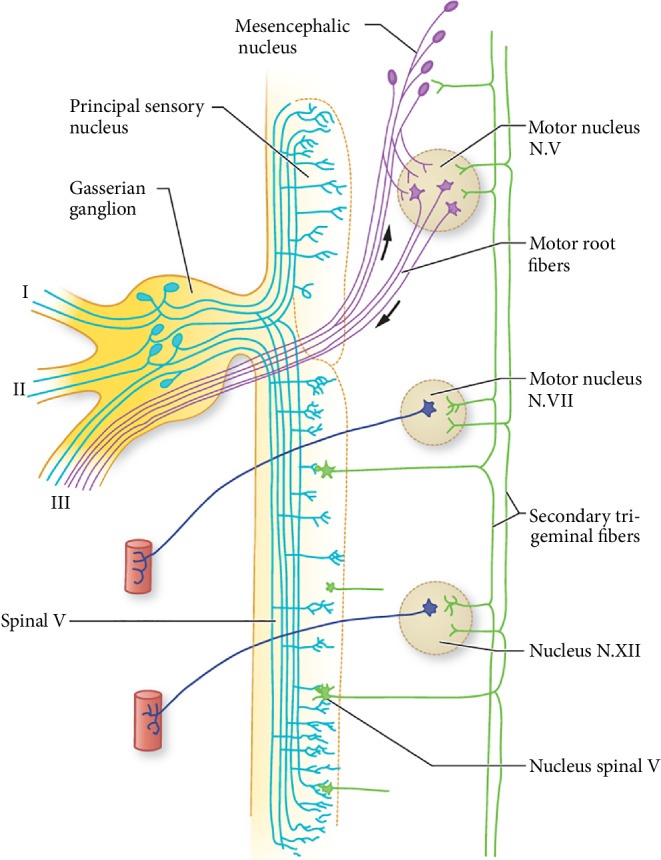
Anatomy of the trigeminal sensory system (adapted from Adams and Victor's Principals of neurology, 10th edition).

Figure 2 illustrates the anatomy of the trigeminal sensory system. Cell bodies of the sensory nerves lie within the Gasserian ganglion. The central axons form the dorsal sensory root which extends into the brainstem and divides into the short ascending and long descending fibers. The former carries tactile, light pressure, and proprioceptive afferents, while the latter carries pain and temperature afferents and extends to the upper cervical segments, which is supported by the evidence of facial pain with medullary trigeminal tracheotomy [6]. From both the principal sensory and spinal trigeminal nuclei, second order fibers form the trigemino-thalamic tract which crosses to the contralateral thalamus. The peripheral fibers of the Gasserian ganglion form the three sensory divisions of the trigeminal nerve. The motor division of the trigeminal nerve arises at the motor nucleus in the mid pons and joins the mandibular division, but does not traverse through the ganglion. This explains motor function sparing with dorsal root compression. As reported by Fisher, pain is caused by a lesion affecting the cell bodies of the ganglion, whereas pressure on the axon typically causes isolated paresthesia [7].

In addition to ischemia, demyelination of the central trigeminal pathways may result in development of aberrant electrical impulses and alternatively irritation of the trigeminal structures creating an area with abnormal excitability [8].

Fitzek et al. evaluated the development and pathophysiology of facial pain following dorsolateral medullary infarct [9]. Eight of the 12 patients developed post-stroke pain within 12 days to 24 months following stroke. The findings on MRI supported the correlation of facial pain with lower medullary lesions, including infarcts affecting the spinal trigeminal tract and/or nucleus.

Ordás et al. reported a case of facial pain in the region supplied by the second division of the trigeminal nerve in a patient with right lateral medullary infarction secondary to dissection of the right vertebral artery [10]. A case of TN resulting from an infarction of the root entry zone of the trigeminal nerve was related by Golby et al. [8]. John et al. reported a case of a 73-year-old woman who developed brief paroxysms of pain on the left lower half of the face—described as sharp, electric shock like—4 days after an ipsilateral lateral medullary infarct [11].

Cases of central pain mimicking TN as a result of pontine ischemia have also been reported since the 1990s [5]. Peker et al. reported sudden onset of facial pain symptoms similar to the classic pain experienced with TN in a patient with a chronic infarct in the left lateral pons [12]. Therefore, the timing between the infarct and development of central pain can vary. There are other cases reported with onset of symptoms several months to years after the infarction [9]. Central facial pain following pontine ischemia can be explained by the anatomical pathway of the trigeminal nerve as described above.

We advise clinicians to be aware of this typical complication of lateral medullary infarct in order to avoid needless imaging and advanced work-up.
